# Use of prescribing safety quality improvement reports in UK general practices: a qualitative assessment

**DOI:** 10.1186/s12913-021-06417-0

**Published:** 2021-04-27

**Authors:** Nada F. Khan, Helen P. Booth, Puja Myles, David Mullett, Arlene Gallagher, Catheryn Evans, Nick Thomas, Janet Valentine

**Affiliations:** 1 Academic Unit of Primary Care, Leeds Institute of Health Sciences, University of Leeds, Level 10, Worsley Building, Clarendon Way, Leeds, LS2 9JT UK; 2 Clinical Practice Research Datalink, Medicines and Healthcare products Regulatory Agency, 10 South Colonnade, Canary Wharf, London, E14 4PU UK; 3 Royal College of General Practitioners, 30 Euston Square, London, NW1 2FB UK

**Keywords:** General practice, Quality improvement, Audit, Prescribing safety

## Abstract

**Background:**

Quality improvement (QI) initiatives are increasingly used to improve the quality of care and reduce prescribing errors. The Royal College of General Practitioners (RCGP) and Clinical Practice Research Datalink (CPRD) QI initiative uses routinely collected electronic primary care data to provide bespoke practice-level reports on prescribing safety. The aim of this study was to explore how the QI reports were used, barriers and facilitators to use, long-term culture change and perceived impact on patient care and practices systems as a result of receiving the reports.

**Methods:**

A qualitative study using purposive sampling of practices contributing to the CPRD, semi-structured interviews and inductive thematic analysis. We interviewed general practitioners, pharmacists, practice managers and research nurses.

**Results:**

We conducted 18 interviews, and organised themes summarising the use of QI reports in practice: *receiving* the report, *facilitators and barriers* to acting upon the reports, *acting* upon the report, and how the reports contribute to a *quality culture*. Effective dissemination of reports, and a positive attitude to audit and the perceived relevance of the clinical topic facilitated use. Lack of time and failure to see or act upon the reports meant they were not used. Factors influencing use of the reports included the structure of the report, ease of identifying cases, and perceptions about coding accuracy. GPs and pharmacists used the reports to conduct case reviews and directly contact patients to discuss unsafe prescribing and patient medication preferences. Finally, the reports contributed to the development of a quality culture within practices through promoting audit activity and acting as a reminder of good prescribing behaviours, promoting future patient safety initiatives, contributing to continuing professional development and improving local networks.

**Conclusions:**

This study found the reports facilitated individual case review leading to an enhanced sense of quality culture in practices where they were utilised. Our findings demonstrate that the reports were generally considered useful and have been used to support patient safety and clinical practice in specific cases.

**Supplementary Information:**

The online version contains supplementary material available at 10.1186/s12913-021-06417-0.

## Introduction

Implementation of clinical guidelines can improve consistency of care and draw attention to ineffective and unsafe clinical practice [1]. Quality improvement (QI) initiatives use an evidence based-approach to actively implement clinical guidelines to maximise use of systems and organisations to deliver better outcomes for patients [2, 3]. Audit and practice feedback programmes are a common QI approach in general practice, and can be used to develop a feedback loop to motivate clinical change and address gaps between current and ideal care [4].

Audit and feedback QI initiatives generally lead to improvements in quality of clinical care and can reduce high risk prescribing [5, 6]. However, the impact of these initiatives are dependent on how feedback is provided, leading to an ongoing need to understand how to optimise effectiveness in different settings [7, 8].

In 2016 the Royal College of General Practitioners (RCGP) and Clinical Practice Research Datalink (CPRD) developed a prescribing safety quality improvement tool to provide reports to each CPRD practice. These reports provided patient-level feedback on six different clinical topics: prescribing of anti-psychotics or anti-depressants in learning disability or autism, sodium valproate in women of childbearing potential, use of Glitazones and non-steroidal anti-inflammatory drugs in heart failure, use of non-steroidal anti-inflammatory drugs in chronic kidney disease, and aspirin monotherapy in patients with atrial fibrillation [9]. The aim of this study was to explore the use of these reports using qualitative interviews. Specifically, we aimed to explore whether and how the QI report was used, barriers and facilitators to use, long-term culture change and perceived impact on patient care and practices systems as a result of receiving the reports.

## Methods

### Interview recruitment

All 1858 practices contributing to CPRD in March 2020 were invited by email to participate in the interview study. We used purposive sampling aiming for a sample of between 8 and 20 interviewees with maximum variation to select respondents from a variety of practices (based on use of the reports, location, list size, region, rural/urban location) and roles (GP, practice manager, pharmacist, nurse) within the practice.

### The RCGP-CPRD quality improvement reports

Details of how the RCGP-CPRD QI reports were developed and selection of quality indicators have been previously published (see Table 1 for a summary of the clinical topics included in previous reports) [9]. The reports are generated using an automated process and are emailed to each individual practice contributing to the CPRD.

**Table 1:** Summary of QI reports distributed to practices

Clinical topic	Background
Long-term prescribing of anti-psychotics or anti-depressants to adults with learning disabilities, autism or both	Based on NHS England’s Stopping Over-Medication of People with a Learning Disability, Autism or Both (STOMP) project
Prescribing of valproate to women of childbearing potential	Developed to support GPs in implementing regulatory recommendations on use of valproate
Prescribing of Glitazones to patients with heart failure	Indicators from the Royal College of General Practitioners (RCGP) Patient Safety Toolkit
Prescribing of non-steroidal anti-inflammatory drugs (NSAIDs) to patients with heart failure	
Prescribing of non-steroidal anti-inflammatory drugs (NSAIDs) to patients with chronic kidney disease (CKD)	
Aspirin monotherapy for stroke prevention in patients with atrial fibrillation (AF)	Suggested by the National Institute for Health and Care Excellence (NICE)

### Interviews

We used a semi-structured interview guide (Additional File 1), developed by AG and DM with open-ended questions to explore how the QI reports were used, perceived barriers and what, if anything, changed in the practices as a result. This allowed the interviewer flexibility in encouraging the participants to talk openly and probe issues raised by the participant [10]. Interviews were conducted by two researchers (DM and CE). DM worked as the RCGP Champion for the RCGP-CPRD QI project at the time of the interviews, and CE works for CPRD to recruit practices to the dataset, therefore some participants had established relationships with both interviewees. Interviews were conducted via video call or telephone and audio-recorded with the interviewee’s verbal permission, and were transcribed verbatim.

### Qualitative analysis

We used an inductive thematic approach to allow generation of codes and themes from the interview data itself [11]. All interviews were coded thematically using NVivo 12 qualitative analysis software by one researcher (NK). Most thematic categories were labelled using descriptive terms grounded in the narratives, whereas others were driven by questions on the interview schedule.

NK reorganised initial themes into coding categories based on the relationships between the themes. There were no new emergent themes within later interviews, so no further interviews were conducted. A second researcher (HB) reviewed the initial coding. Themes were re-reviewed and re-coded as an iterative process, and a model was developed to represent the key themes. Results are illustrated with verbatim quotes.

Please see Additional File 2 for a full ‘Consolidated criteria for reporting qualitative research’ (COREQ) checklist [12].

## Results

We received 30 responses to the initial email to CPRD practices, from which we drew a purposive sample for interview as described previously. We conducted 18 interviews with practice staff working in a range of roles within 18 different GP practices (see Table 2). Interviews were conducted between March and July 2020.

**Table 2:** Description of interviewees

Practice number	Role in the practice	List size	Urban/rural	Location	Used report?
1	GP partner	12000	Suburban	Midlands and East	No
2	Salaried GP	20000	Urban	Midlands and East	Yes
3	Practice manager	17500	Rural/urban	Midlands and East	Yes
4	GP partner	9000	Urban	Scotland	Yes
5	GP partner	8800	Semi-rural	Scotland	Yes
6	Business manager^a^	11000	Rural/urban	Scotland	Yes
7	Pharmacist	15000	Urban	London	Yes
8	GP partner	12998	Urban	North	No
9	GP partner	8000	Urban/suburban	North	Yes
10	Pharmacist	25000	Urban	South	Yes
11	GP partner	12000	Urban	Midlands and East	Yes
12	GP partner	2500	Rural	North	Yes
13	Pharmacist	7500	Rural/urban	Wales	Yes
14	Research nurse	10000	Urban	Midlands and East	Yes
15	Pharmacist	9676	Urban	London	Yes
16	Practice manager	8000	Urban	North	Yes
17	GP partner	15500	Suburban	South	Yes
18	GP partner	18500	Urban	Midlands and East	No

^a^Similar role to practice manager

### Themes

**Fig. 1:**
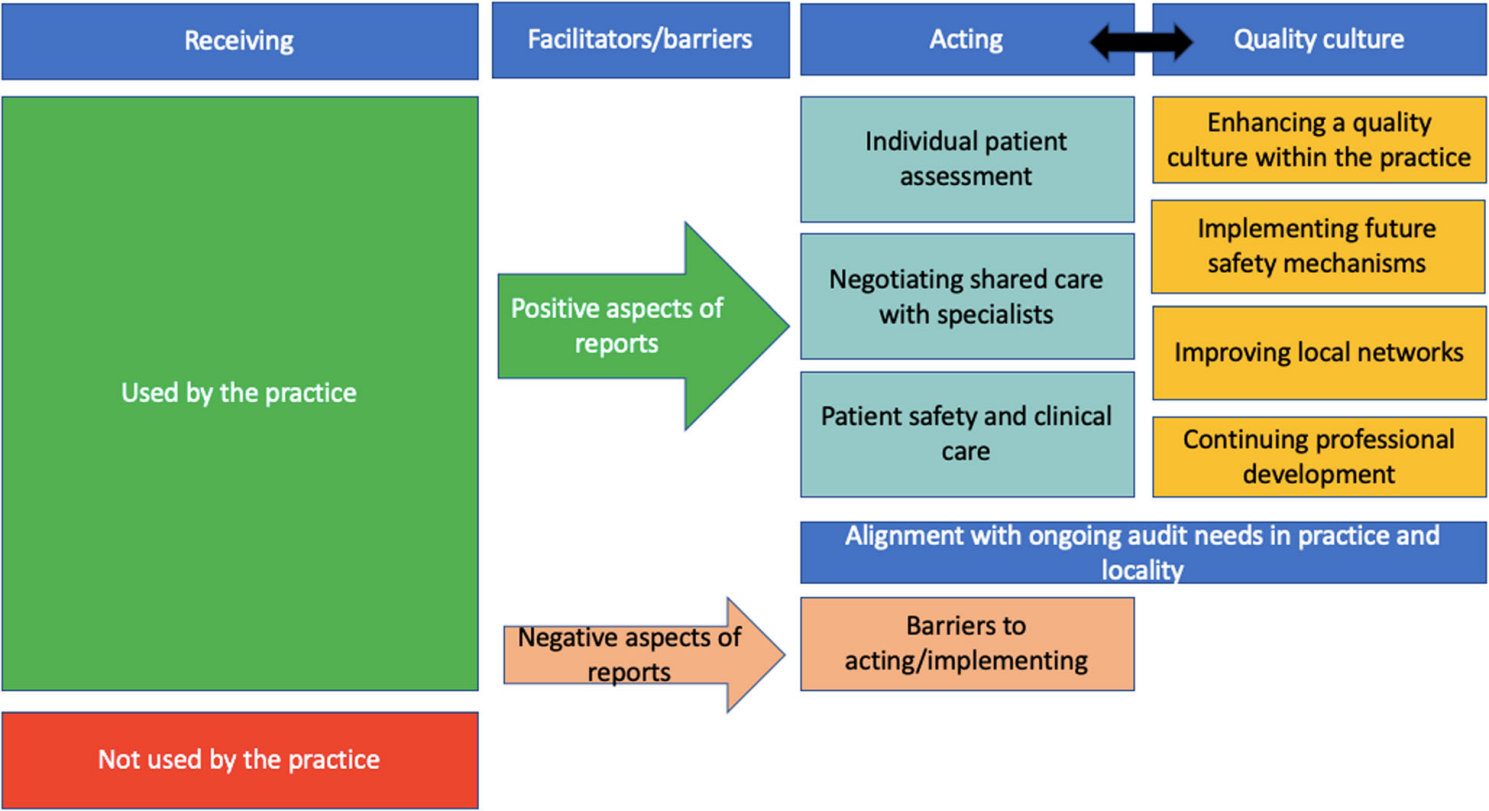
Coding framework

We present the key themes in a model summarising the use of the QI reports within the practices: *receiving* the report, *facilitators and barriers* to acting upon the reports, *acting* upon the report within the practice, and how the reports contribute to a *quality culture* (Fig. 1).

### Receiving the report

Interviewees described how the reports were received at the practice, and how practice attributes and report characteristics influenced whether they were used.

The reports were typically sent to a recipient nominated by the practice; frequently a lead GP or practice manager. In practices where the reports were utilised, the reports were redistributed within the practice and sent onwards either to the prescribing or clinical lead in the practice or the patient’s listed GP for action:

*‘Everything tended to go through one point and then was disseminated, so we had one GP that was looking after the QOF heart things, so I think the reports would go through to them and I was the learning disability person so that would come through to me We had a pharmacist, so anything prescribing would go through her first’* [Practice 2, report user, GP]

Another avenue for dissemination of the reports within the practice was a discussion within a practice or prescribing meeting on how to action the recommendations, or to highlight prescribing queries with individual GPs:

‘*Sometimes, it [the QI report] is discussed in the practice meeting as which way we want to handle this and the GPs provide guidance, ‘OK, so let it go to [name of GP]’, or if there are any concerns or anything or any clarification needed I can pass it back off to the doctors*’ [Practice 15, report user, pharmacist]

Several respondents discussed how having a practice-based pharmacist facilitated use of the QI report recommendations as they were able to review the clinical guidance to determine which action needed to be taken, replicate audit searches and contact patients to organise an appointment or medication review. Including the prescribing team also ensured that the report was not lost amongst the bulk of daily emails:

‘*The other thing I think would be really useful, for us anyway, is cc’ing our prescribing team into those reports. They all come to me, which is fine but I have hundreds of emails every day and then if it is just coming to one person then it can just sit there*.’ [Practice 18, report non-user , GP]

In two practices, the reports went to a lead GP partner who forwarded the report on to someone else in the practice. However, it was unclear how the report was then dealt with. One GP described this as a missed opportunity,

‘*I think it’s partly my fault [that the report was not used] because I printed off the reports and gave them to our prescribing team, but I don’t think they fully realised what they were, and then when I checked with them and asked ‘do we use these’ about three months ago, [prescribing lead] said ‘Gosh, these are brilliant. I haven’t used these as much as I could have done’'* [Practice 18, report non-user, GP]

QI report emails tended to be looked at, or used, when interviewees placed a pre-conceived value upon the reports for their clinical importance or in terms of the severity of the prescribing safety issue. Interviewees who valued audit activities liked receiving reports, finding them useful and interesting. One research nurse commented that,

*‘I find them endlessly fascinating – I like doing audit. To help your practice, and your patients – they’re a really, really good thing.’* [Practice 14, report user, research nurse]

Reports that aligned with practice, QOF, MHRA reporting or CCG audit needs were more likely to be utilised by some interviewees. As one pharmacist noted:

‘*A lot of what comes up in these reports are things that our local health board has on patient safety indicators … so they were definitely on our radar but because we got the report it actually prompted us to do more structured action.’* [Practice 13, report user, pharmacist].

However, in some practices doing similar audits and reports using the same clinical indicators, the QI reports were not looked at as they replicated work that was already being done.

The QI reports were not utilised when the recipient did not see the report or did not have time or capacity to deal with it. One GP reported,

‘*I mean, it is useful, I just wish I personally had the time to, and one of my partners, that we could look into these things because it is practice based data, isn’t it?*’ [Practice 8, report non-user, GP].

### Facilitators and barriers to acting upon the reports

Interviewees described several facilitators and barriers to acting upon the reports. One advantage of the RCGP-CPRD QI reports compared to other audit and feedback reports was that they provided patient-level feedback and allowed re-identification of patients for case-finding as reported by one GP:

‘*The advantage we get with your reports is actually it tells me the patients and I’ve got the EMIS numbers so they’re actually easier to use and make a difference … you get that list of EMIS numbers [a patient identifier within a primary care clinical system] and I will look at ‘patient 2441’ and look through their notes and ask ‘can I make a difference*’ [Practice 11, report user, GP]

Respondents discussed the layout and structure of information within the report. Interviewees preferred an actionable patient list early on within the text of the report, which captured and focussed attention on the patients needing review. Conversely, reports that were not structured in a user-friendly manner were seen as a barrier to quickly interpreting their clinical usefulness. In particular, two interviewees did not like having a large amount of contextual background early on within the report:

‘*I don’t want to read through all the justification for the targets while I’m trying to look for my data. So, I don’t want to have to read through three paragraphs and then get how I did. I want how I did to be there and then the other stuff could be in an appendix or a hyperlink or something.*’ [Practice 9, report user, GP]

Respondents discussed other barriers to acting upon the reports effectively. One perceived barrier was a concern about coding accuracy of diagnoses within the practice as reported by one GP:

‘*The next one for aspirin [as monotherapy for atrial fibrillation (AF)], I left that for a year as I just couldn’t bring myself to look at it because I knew fully well a lot of them would be patients that had AF coded during an admission years ago and it’s now resolved … or they had AF related to sepsis or infection or something, so I actually got our trainee to do a QI project on that and she’s gone through all of them and actually for most of them it was AF resolved, they didn’t actually have AF, it was a coding issue.*’ [Practice 5, report user, GP]

Patients screened in previous audit cycles on the same quality indicator were not excluded or highlighted in subsequent searches, for instance, if patients were previously deemed to be on medication appropriately. Two GPs felt that this was the most ‘off-putting’ feature of the reports and led to a loss of interest in looking at serial versions of the report. One GP suggested use of a simple ‘tool’ such as an Excel sheet to allow review of old and new cases in order to flag up new patients that needed action, and exclude patients that had already been checked. Similarly, it was seen as time-consuming when reports did not exclude suitable prescribing behaviours, for instance, not excluding women with a history of hysterectomy or sterilisation from the sodium valproate report. As one GP commented,

‘*as the practitioner doing the review, I don’t want that. I want a high hit rate because if you have ten to review and the first four have had a hysterectomy, you’re gonna lose interest.’* [Practice 9, report user, GP].

### Acting upon the reports

#### Individual patient assessment

Once the reports were disseminated through the practice, GPs acted upon the report by reviewing patient case notes, or directly contacting patients to discuss the prescribing query. This practice business manager describes how patients were contacted as a result of the sodium valproate QI report,

*The information I see in your reports, such as the valproate one, I do remember we used the information that came through from you quite significantly to get in touch with all our patients that were of childbearing age and make sure … so, it allowed us to run an audit and make sure that all those patients were contacted and either advised or removed from that medication. So, yes, the safety side of it is very useful. [Practice 6, report user, Business manager]*

Pharmacists were also involved in reviewing individual patient risk factors, such as reduced renal function in prescribing for NSAIDs, and discussed medication dosage reduction or modification with a GP for action. In some cases, patient notes were reviewed and it was determined that no further action was required. One GP noted that

‘*there was so little action needed actually on them, when I did the case reviews … generally when people were on a potentially unsafe combination, or when I’d looked at the patients with a learning disability, they generally had a dual diagnosis or somebody was already on the case with a reduction plan.*’ [Practice 9, report user, GP].

Clinicians also used the QI reports to explore patient preference in continuing or stopping the medication based on the safety information as described by one GP:

‘*There were probably two or three, from looking at the notes, we pulled in to have a chat with their medication and I think possibly one may have stopped their anti-inflammatories [in the context of chronic kidney disease] and two carried on, so it triggered a review process with those ones once I’d had a look through the notes. So, obviously this brings up a discussion point with the patient to highlight the risk whether it’s something they want to carry on doing*.’ [Practice 11, report user, GP]

#### Shared care with secondary care prescribers

Some medications included in the QI reports, including sodium valproate and anti-psychotics, were often initiated by a secondary care specialist team, and the primary care clinician had to negotiate issues around prescribing safety with the specialist. In some cases, the specialist team was contacted to discuss the medication, however, this was sometimes viewed as a challenge,

*‘The biggest difficulty that we have with antipsychotics generally is that it’s not our thought that this patient should have an antipsychotic. Some specialist has suggested it and we’re responding to that … this is a bit of a burden as you do bear the responsibility for these prescriptions, but you don’t really control whether the patient should have them or not’* [Practice 4, report user, GP]

Although the QI report might highlight a prescribing safety issue, some GPs sometimes felt disempowered to make changes to medications initiated by secondary care. The same GP discussed ‘pushback’ from secondary care prescribers,

‘*suggesting I would like to stop this patient with learning disability’s antipsychotics; you tend to get a lot of pushback against it. Similarly with dementia, a lot of that is specialist initiated and I’m always getting involved with arguments*.’ [Practice 4, report user, GP].

#### Patient safety and clinical care

Interviewees discussed how the QI reports supported patient safety, and welcomed the reports as a tool to ensure and highlight safe prescribing behaviours. One common theme across interviewees was the use of the QI reports as a safety net to identify instances of unsafe prescribing ‘slipping through the net’,

‘*With the patient safety element we’ve got to get to a point where prescribing errors are always going to occur, so actually having a system that picks up those errors as we are going … I think my partners take this audit process as a bit of reassurance that there is somebody, there is a safety process around that, but whether it’s absolutely changing their behaviours I don’t know. I just think it might be reassuring them that there is a process that is making sure that errors are picked up*’ [Practice 11, report user, GP].

Reports were sometimes discussed in practice or prescribing meetings and used to identify safety issues. Specifically, the reports were sometimes used to highlight individual GP prescribing patterns or clinical areas where prescribing was suboptimal. One pharmacist found it empowering to have the QI reports, as it enabled them to discuss prescribing issues with GPs and helped negotiate a perceived power imbalance,

*‘ … having that piece of paper when talking to that particular prescriber would be really helpful, saying I’m not that baddie, it’s them. Do you know what I mean? Sometimes it takes away the tension … sometimes these conversations could be quite difficult, saying ‘your practice in this particular [area] isn’t what we would exactly like it to be’ … so sometimes shifting the blame is not a bad thing!’* [Practice 13, report user, Pharmacist]

### Quality culture

#### Enhancing a quality culture within the practice

Interviewees described how receiving the QI reports enhanced the quality improvement culture within the practice. Reports often triggered more practice audit activity as clinicians and pharmacists built up robust searches based on the quality indicator topics to ensure that patients were not on unsafe medications.

The QI reports also increased clinician vigilance relating to prescribing safety, and planted a seed in the mind of clinicians to think about safety issues when prescribing risky medications in the future,

*‘So, once we have seen those clinic letters, it … rings a bell and you know to check immediately, ‘has this been put in place’ [risk assessment]? Or maybe they have been discharged from a mental health hospital and just before you add it on, it rings a bell, ‘oh I need to check this, is this in place?’ So I think this type of quality improvement does help’.* [Practice 15, report user, pharmacist]

Similarly, the QI reports served as a reminder of good prescribing behaviour,

*‘I think that they are a good reminder because even though we know we shouldn’t prescribe NSAIDs for CKD, sometimes you might think, ‘OK it’s quite a young patient ‘ but it’s a good reminder to check the kidney function first’* [Practice 13, report user, pharmacist]

#### Prescribing safety initiatives

The QI reports acted as a trigger to implement future safety mechanisms to reduce unsafe prescribing. One approach to minimise long-term risky prescribing was to take patients off repeat medication. This triggered a review process by the GP the next time it was requested by the patient:

*‘The people that had had it for a while [NSAIDs in CKD], I took it off their repeat screen so that would prompt the GP to look at it next time they requested it. And also I put notes on all of them just to confirm that I had looked at them … it was a little bit of a reminder for [the GP] to consider what they were doing when, if they were planning to prescribe it again’* [Practice 10, report user, pharmacist]

#### Improving local networks and alignment with practice and local audit needs

Some GPs found the reports helpful in promoting prescribing safety and wanted similar QI reports at Primary Care Network (PCN) level across several different practices in the area. Interviewees described the need for integrated audit across local networks.

Some practices were doing similar audit work, and the QI reports were seen as complementary to practice-based audits. In particular, several practices were working on locally and nationally driven projects on NSAID prescribing in CKD and receiving a QI report on the same topic was seen as beneficial and complementary to their work.

‘*It does overlap with quite a lot of the work we’re doing for [local county] … for the prescribing incentive scheme … one of them nationally, we are doing an audit on non-steroidals anyway, which is great because this actually overlapped a lot with that … so actually some of the themes were ones we’d looked at fairly recently or we’re working on at the moment. So that was good because you know it certainly overlapped with the two projects.*’ [Practice 10, report user, pharmacist]

#### Continuing professional development

Reports were seen by one GP as feeding into continuing professional development and were used as an adjunct to other learning resources.

‘*We try to keep up-to-date, but the reality is that some things do pass you by, so I know there were people out there who still didn’t realise not to carry on with Glitazones when people do get heart failure, so in terms of that it [the QI report] changes, it helps you update and therefore it changes your future practice*.’ [Practice 12, report user, GP]

GPs also used the process of going through the QI reports as part of their annual appraisals and revalidation,

‘*I’ve read them and used them on numerous occasions, and I’ve used them for my revalidation as well.*’ [Practice 11, report user, GP]

## Discussion

### Summary of principal findings

This qualitative study has explored general practice staff perspectives on the use and perceived impact of the RCGP-CPRD QI reports. Effective dissemination of QI reports in the practices enables their use, whilst lack of time and low visibility in busy email inboxes meant that the reports were not seen or used. The case-finding and clear data-led report layout were seen as specific benefits of the RCGP-CPRD QI reports.

The reports highlighted to prescribers when a case review or patient contact to discuss medication was warranted, and in some cases led to patients being taken off potentially unsafe medications. Additionally, GPs used the reports to explore prescribing preferences with patients, supporting shared decision-making through discussions about risks and benefits of continuing a medication. Some interviewees found it helpful to know that the reports were in place as a system for safety-netting areas of unsafe prescribing behaviour, especially as clinical work became busier.

As a result of the report, some practices implemented specific safety processes, for instance, removing potentially unsafe medications from the patients’ repeat medication list to minimise prescribing without a clinician review. Finally, the reports enhanced a sense of quality improvement and facilitated audit activity and continuing professional development.

### Comparison with existing literature

Brehaut et al. describe fifteen suggestions for optimising effectiveness of feedback interventions based on their previous experience, and some of the suggestions echo our findings, including recommending actions consistent with established goals and priorities, provision of individual rather than general data and actionable messages followed by optional detail [13]. Payne and Hysong described physician acceptance of audit and feedback, and developed a physician feedback model that looked specifically at feedback acceptance [14]. Their findings, including how physicians accept feedback, actions related to the behaviours physicians engaged in after receiving reports, and impact related to the effect feedback had on patient management, relate to our findings on patient contact and enhancement of a quality culture within the practice. Lastly, Brown et al. synthesised evidence from 65 international qualitative studies to develop a clinical performance feedback intervention theory (CP-FIT) of how feedback and intervention can be optimally designed and implemented [15]. Our findings reflect some of the model elements, including that acceptance and intention were more likely when feedback measured aspects of care that were thought to be clinically meaningful. Importantly, acceptance was further facilitated when feedback recipients could exception report patients in the reports based on clinical judgement. Some of the findings from this research may be applicable to other teams using quality improvement reports in similar healthcare settings.

### Strengths and limitations

This evaluation has included a range of health care professional perspectives and experiences of using the RCGP-CPRD QI reports. We were also able to explore perspectives of those who did not utilise the report in practice.

This is a small study looking at a specific quality improvement project based in UK general practice. We are therefore limited in being able to make broad conclusions on the impact and effectiveness of quality improvement reports in general based on this qualitative study. The strengths are that we have elicited responses from a range of practices, but it is possible that our sample was largely drawn from practice staff who found the reports useful, and our findings may over-represent the level of use of the CPRD QI reports in practice. However, we were able to elicit responses from practices that did not routinely use the QI reports, and these responses contributed to our model development.

### Implications for research and practice/recommendations

Our findings demonstrate that the RCGP-CPRD QI reports were generally considered useful and have been used to improve clinical practice. Respondents discussed barriers and facilitators to dissemination, communication and the content and structure of the report. Recommendations based on the results from this study on how these reports can be further improved are summarised in Table 3, however further work needs to be done before definitive change can be recommended.

**Table 3:** Initial recommendations for audit and feedback and quality improvement reports in primary care

**Dissemination**	Discuss with each practice who the report should be sent to, i.e. prescribing lead
	Actively involve practice based pharmacists – include on email distribution lists
	Suggest that if the report is forwarded onwards by a lead GP/practice manager, the next recipient acknowledges receipt and how the report will be used, if at all
**Communication with practices**	Emphasise that the reports might save practices time on audit activity
**Communication with patients**	Provision of guidance letter and example patient communication with the report
**Communication within local networks**	Include resources and suggestions to encourage practices to discuss how they have used reports within their local primary care networks
**Content/topic**	Aim for clinical content and topics that are in line with current local and national priorities for quality improvement
**Structure of report**	Provide individual patient identifiers if possible to expedite case finding
	Prioritise information on patients needing review at the beginning of the report, avoid lengthy background contextualising the report
	Incorporate a tool to allow flagging of patients included in previous searches, so if they were excluded by a clinician from the quality indicator they dont' have to go back over the same searches/exclusion criteria

## Conclusions

This research has explored the experiences of practices receiving the RCGP-CPRD QI reports. Future research should explore the quantitative impact of the reports in terms of changing prescribing behaviours.

## Supplementary Information

**Additional file 1.** Interview Guide

**Additional file 2.** COREQ checklist

## Data Availability

The datasets generated and/or analysed during the current study are not publicly available due to some personal data contained within the interviews, but may be available from the corresponding author on reasonable request.

## Abbreviations

AF: Atrial fibrillation
CCG: Clinical commissioning group
CKD: Chronic kidney disease
CPRD: Clinical Practice Research Datalink
GP: General practitioner
MHRA: Medicines and Healthcare products Regulatory Agency
NSAID: Non-steroidal anti-inflammatory drugs
PCN: Primary care network
RCGP: Royal College of General Practitioners
QI: Quality improvement
