# Correction to: Use of prescribing safety quality improvement reports in UK general practices: a qualitative assessment

**DOI:** 10.1186/s12913-021-06547-5

**Published:** 2021-06-02

**Authors:** Nada F. Khan, Helen P. Booth, Puja Myles, David Mullett, Arlene Gallagher, Catheryn Evans, Nicholas P. B. Thomas, Janet Valentine

**Affiliations:** 1 Academic Unit of Primary Care, Leeds Institute of Health Sciences, University of Leeds, Level 10, Worsley Building, Clarendon Way, Leeds, LS2 9JT UK; 2 Clinical Practice Research Datalink, Medicines and Healthcare products Regulatory Agency, 10 South Colonnade, Canary Wharf, London, E14 4PU UK; 3 Royal College of General Practitioners, 30 Euston Square, London, NW1 2FB UK

**Correction to: BMC Health Serv Res 21, 394 (2021)**

**https://doi.org/10.1186/s12913-021-06417-0**

In the original publication of this article [1], the spelling of the author Nicholas PB Thomas needs to be corrected.

The incorrect author name is: Nick Thomas

The correct author name is: Nicholas PB Thomas

The author group has been updated above, and the original publication has been corrected.
