# Supplementary material for: Use of prescribing safety quality improvement reports in UK general practices: a qualitative assessment
Source: BMC Health Serv Res. 2021 Apr 27;21:394. doi: 10.1186/s12913-021-06417-0 (PMC8077765; doi:10.1186/s12913-021-06417-0)
Supplement: Supplementary file 1 — Additional file 1. Interview Guide [file 12913_2021_6417_MOESM1_ESM.docx]

**Additional File 1 – Interview guide**

The questions to be covered are as below; mostly open questions with prompting points in square brackets. We will pilot these questions to test both the content and the ability to record the responses before rolling out widely.

| **Question** | **Estimated time** |
| --- | --- |
| My name is… and my role is …  Are you happy for this interview to be recorded?  Thanks for your time today. This is an interview about the Quality Improvement data reports that focus on patient safety and prescribing. This interview is part of a structured evaluation of the project. We’ve set up the interview today because we would like understand if the reports have had an impact, and get feedback that informs the future development of the project.  Can you confirm your role at the practice? |  |
| What happened at your practice as a result of receiving the reports?  Users: [Is the report shared at the practice? What steps are then taken? Is there a lead GP who takes responsibility? Are reviews discussed in practice meetings?]  Non-users: [What were the barriers? What would make you use the reports in future?] |  |
| What did you or your colleagues do as a result?  [Did you change the treatment of any patients as a result of receiving the reports? How? (Use list of indicators to prompt if necessary)]  [We are looking to compile anonymous case studies about the reports. Can you give us details of individual treatment that could be written up this way? I.e. description of patient; safety issue identified by report; action taken; benefit to patient]  [Like all QI initiatives, these reports are designed to change behaviour. Have you reviewed or changed how you prescribe at your practice in the longer term?] |  |
| We are reviewing the frequency and content of the reports, considering retiring some of the indicators and adding new ones. Your answers will help us to make plans for the future; thank you for your time today. |  |
