# Supplementary material for: Use of prescribing safety quality improvement reports in UK general practices: a qualitative assessment
Source: BMC Health Serv Res. 2021 Apr 27;21:394. doi: 10.1186/s12913-021-06417-0 (PMC8077765; doi:10.1186/s12913-021-06417-0)
Supplement: Supplementary file 2 — Additional file 2. COREQ checklist [file 12913_2021_6417_MOESM2_ESM.docx]

**Additional File 2 - COREQ Checklist**

| **Personal characteristics** | | |
| --- | --- | --- |
|  | Interviewer/facilitator  Which author/s were involved in data collection? | DM and CE |
|  | Credentials  What were the researchers’ credentials? | NK: MSc, DPhil, MBBS  HB: DPhil  DM: MA  CE: MSc  PM: BDS, MPH, PhD, FFPH  JV: BSc(Hons), PhD |
|  | Occupation  What was their occupation at the time of the study? | NK: NIHR Academic Clinical Fellow in General Practice  HB: Senior Researcher - CPRD  DM: - RCGP Champion for patient safety and prescribing  CE: GP Engagement Manager - CPRD  PM: Head of Observational Research CPRD  JV: CPRD Director |
|  | Gender  Was the researcher male or female? | NK: Female  HB: Female  DM: Male  CE: Female  PM: Female  JV: Female |
|  | Experience and training  What experience or training did the researchers have? | NK has planned and conducted several qualitative studies, including conducting in-depth qualitative interviews and analysis.  DM has contributed to systematic reviews of behaviour change interventions in prescribing and patient safety, as well as to quantitative studies. He interviewed and surveyed users of the reports on a regular basis as part of the process of continuous improvement of the reports.  PM has led and conducted several qualitative studies previously including study design, interviews, analysis and write-up |
| **Relationship with participants** | | |
|  | Relationship established  Was a relationship established prior to study commencement? | Some participants had long-established relationships with DM and CE, as they had been involved in the project from the initial development of the reports. For others, there was no previous relationship. This spread was a deliberate aim of our sampling strategy. |
|  | Participant knowledge of the interviewer  What did the participants know about the researcher? E.g. personal goals, reasons for doing the research | Interviewees were told the role of the interviewer at the beginning of the interview, and were told that the reason for doing the research was to understand if the reports have had an impact, and get feedback that informs the future development of the project. They were also asked for their permission to be recorded and for their words to be used. |
|  | Interviewer characteristics – what characteristics were reported about the interviewer? | DM was the RCGP Champion for the RCGP-CPRD QI project. No other characteristics were reported; the interviewer did not turn on his video during the interviews, so characteristics were not observable by participants. |
| **Theoretical framework** | | |
|  | Methodological orientation and theory  What methodological orientation was stated to underpin the study? e.g. grounded theory, discourse analysis, ethnography, phenomenology, content analysis | Inductive thematic analysis |
|  | Sampling  How were participants selected? | Purposive |
|  | Method of approach  How were participants approached? | Email to all participating practices in the CPRD |
|  | Sample size  How many participants were in the study? | 18 |
|  | Non‐participation How many people refused to participate or dropped out? Reasons? | 5 more interviews were booked in but didn’t go ahead. This was largely due to practices becoming busier because of covid-19. 1 interview didn’t happen because of a family bereavement (interviewee). |
| **Setting** | | |
|  | Setting of data collection  Where were the data collected? e.g. home, clinic, workplace | About half of the interviews took place at the interviewees home and half at practice offices. Interviewers were working from home. |
|  | Presence of non‐participants  Was anyone else present besides the participants and researchers? | No |
|  | Description of sample  What are the important characteristics of the sample? e.g. demographic data, date | Role within the practice, location of practice |
| **Data collection** | | |
|  | Interview guide Were questions, prompts, guides provided by the authors? Was it pilot tested? | An interview guide was used (see Appendix 1). Within the research team, DM & CE carried out a test interview; it was not tested on practices. |
|  | Repeat interviews Were repeat interviews carried out? If yes, how many? | No |
|  | Audio/visual recording Did the research use audio or visual recording to collect the data? | Both. |
|  | Field notes Were field notes made during and/or after the interview or focus group? | For one interview, which had to be done by phone and where recording was therefore not available. |
|  | Duration What was the duration of the interviews or focus group? | From 20 minutes to one hour. The variation was due to (1) participant contribution (some had much more to say than others) and (2) participant availability (pressure of time was a particular issue due to the Covid-19 pandemic). |
|  | Data saturation  Was data saturation discussed? | Yes, during our analysis and data workshop |
|  | Transcripts returned  Were transcripts returned to participants for comment and/or correction? | No |
| **Data analysis** | | |
|  | Number of data coders  How many data coders coded the data? | NK coded the data. NK, HB, CE and KL were involved were developing the coding framework |
|  | Description of the coding tree  Did authors provide a description of the coding tree? | Yes |
|  | Derivations of themes  Were themes identified in advance or derived from the data? | Derived from the data |
|  | Software  What software, if applicable, was used to manage the data? | NVivo 12 |
|  | Participant checking  Did participants provide feedback on the findings? | No |
| **Reporting** | | |
|  | Quotations presented  Were participant quotations presented to illustrate the themes/findings? Was each quotation identified? | Yes |
|  | Data and findings consistent  Was there consistency between the data presented and the findings? | Yes |
|  | Clarity of major themes  Were major themes clearly presented in the findings? | Yes |
|  | Clarity of minor themes  Is there a description of diverse cases or discussion of minor themes? | Yes |
